# Association of Urban-Rural Health Insurance Integration With Health Outcomes Among Middle-aged and Older Adults in Rural China

**DOI:** 10.1001/jamanetworkopen.2023.7583

**Published:** 2023-04-04

**Authors:** Xin Ye, Yanshang Wang

**Affiliations:** 1 Institute for Global Public Policy, Fudan University, Shanghai, China; 2 LSE-Fudan Research Centre for Global Public Policy, Fudan University, Shanghai, China; 3 School of Public Health, Peking University, Beijing, China; 4 China Center for Health Development Studies, Peking University, Beijing, China

## Abstract

This cohort study examines the association of urban-rural health insurance integration with health outcomes among middle-aged and older adults in rural China.

## Introduction

Social health insurance is generally considered an important means of achieving universal health coverage, especially for low-income and middle-income countries.^1^ China’s urban-rural health insurance integration is a very important institutional reform to address inefficiency of the health insurance system and related health inequalities.^2^ However, its associations with a broad range of health outcomes have not been explored yet. This cohort study aims to examine the associations of the integration with health outcomes among middle-aged and older rural adults and possible mechanisms behind those associations.

## Methods

Data came from the China Health and Retirement Longitudinal Study (2011-2018). This study followed the Strengthening the Reporting of Observational Studies in Epidemiology (STROBE) reporting guideline and received institutional review board approval from Peking University. All participants provided written informed consent. We focused on 54 934 middle-aged and older adults with rural household registration, who would be most affected by the integration policy.^3^ Participants enrolled in the New Rural Cooperative Medical Scheme, Urban Resident Basic Medical Insurance, or Urban and Rural Resident Basic Medical Insurance were then included (50 527 participants). Furthermore, we retained 48 913 respondents without missing values in key covariates, who constituted the final panel.

The independent variable, urban-rural health insurance integration, indicated whether a province implemented the integration policy in the corresponding survey year. On the basis of the staggered implementation of the integration across cities over time, we used a staggered difference-in-differences (DID) model to examine the associations. A multiperiod DID model (event study model) was used to test the parallel trend hypothesis (eAppendix in Supplement 1). Data analysis was performed from September 2022 to February 2023 using Stata SE statistical software version 16 (StataCorp).

## Results

At baseline, 12 072 participants (mean [SD] age, 58.78 [0.09] years; 5775 men [47.8%]) were included. The proportion of participants who experienced the urban-rural health insurance integration increased from 1.2% (142 participants) in 2011 to 93.0% (11 920 participants) in 2018 (Table 1). The integration was significantly associated with better self-rated health (DID, 0.05; 95% CI, 0.01 to 0.09) and fewer depressive symptoms (DID, −0.03; 95% CI, −0.05 to −0.01) for middle-aged and older rural adults. The results were robust when excluding 1031 individuals living in the 4 provincial-level municipalities who had different socioeconomic backgrounds (Table 2).

**Table 1.** Summary Characteristics of Participants at 4 Waves of the China Health and Retirement Longitudinal Survey

Characteristic	Respondents, No. (%)			
	2011 (n = 12 072)	2013 (n = 12 795)	2015 (n = 11 231)	2018 (n = 12 815)
Independent variable: integrated insurance	142 (1.2)	1008 (7.9)	2358 (21.0)	11 920 (93.0)
Outcome variables				
Self-rated health status (reference, poor)				
Fair	4024 (46.9)	6417 (52.4)	5702 (53.7)	5686 (47.8)
Good	2222 (25.9)	2910 (23.8)	2425 (22.9)	2671 (22.5)
Impaired ADLs	2173 (18.2)	2348 (18.5)	2397 (21.8)	2666 (21.0)
Impaired instrumental ADLs	2823 (23.4)	3364 (26.4)	3004 (26.8)	3568 (27.9)
Chronic diseases, mean (SD), No.^a^	1.33 (0.01)	1.46 (0.01)	1.83 (0.02)	2.16 (0.02)
Depressive symptoms	4493 (40.6)	3980 (34.3)	3836 (36.2)	4756 (40.6)
Cognitive scores, mean (SD)^b^	10.05 (0.04)	10.28 (0.04)	10.13 (0.04)	9.15 (0.05)
Key covariates				
Age, mean (SD), y	58.78 (0.09)	59.60 (0.09)	60.38 (0.09)	62.33 (0.09)
Sex				
Male	5775 (47.8)	6033 (47.2)	5296 (47.2)	5867 (45.8)
Female	6297 (52.2)	6762 (52.9)	5935 (52.8)	6948 (54.2)
Married	10 548 (87.4)	11 157 (87.2)	9727 (86.6)	10 861 (84.8)
Education (reference, illiterate)				
Primary school	2687 (22.3)	2919 (22.8)	2853 (25.4)	3312 (25.8)
Secondary school and above	2983 (24.7)	3217 (25.1)	2762 (24.6)	3206 (25.0)

Abbreviation: ADL, activities of daily living.

^a^ The number of chronic conditions ranged from 0 to 13.

^b^ Scores ranged from 0 to 21, with higher scores indicating better cognitive function.

**Table 2.** Estimated Difference-in-Differences in the Associations Between Urban-Rural Health Insurance Integration and Health Outcomes for Middle-aged and Older People in Rural China

Variable	Estimated difference-in-differences (95% CI)					
	Better self-rated health	Impaired ADLs	Impaired instrumental ADLs	Chronic diseases	Depressive symptoms	Cognitive scores
Whole sample	0.05 (0.01 to 0.09)	−0.01 (−0.04 to 0.02)	0.001 (−0.03 to 0.03)	0.04 (−0.05 to 0.13)	−0.03 (−0.05 to −0.01)	0.22 (−0.08 to 0.51)
Robustness test excluding those from Beijing to Tianjin, Shanghai, and Chongqing	0.05 (0.01 to 0.09)	−0.01 (−0.04 to 0.02)	0.001 (−0.03 to 0.04)	0.04 (−0.05 to 0.14)	−0.02 (−0.04 to −0.01)	0.29 (−0.03 to 0.62)

Abbreviation: ADL, activities of daily living.

The possible mechanisms behind the association between the integration and the outcomes could be that the integration increased the probability of having inpatient services (DID, 0.03; 95% CI, 0.01 to 0.04) and reduced both out-of-pocket inpatient costs (DID, −0.11; 95% CI, −0.19 to −0.02) and the incidence of not being hospitalized when needing inpatient care (DID, −0.15; 95% CI, −0.25 to −0.05). These positive health outcomes were particularly salient for people living in rural areas, those who were far from their children, and those from households with poor economic status (the lowest one-third of household consumption).

## Discussion

This cohort study is one of the first to comprehensively examine the health benefits of the urban-rural health insurance integration in China. The findings suggest that the integration improved only certain health outcomes, especially for those who were economically disadvantaged. The possible mechanisms behind this improvement could be through increased inpatient services utilization, reduced out-of-pocket inpatient costs, and reduced underutilization of hospitalization. Our findings were consistent with previous literature^4,5,6^ documenting no or moderate associations of China’s health insurance programs with health outcomes. The findings may be attributed to the short time period of our study, or they may reflect the quality and efficiency problems inherent in the health care system.

Overall, through an examination of the health insurance integration in China, our study provides new evidence for the associations between health insurance and health outcomes that will offer nuanced insights for policy makers to further improve health policy. However, we only observed short-term associations and did not fully capture the actual progress and the genuine benefits of the integration. Future research should pay closer attention to the long-term health outcomes of the health insurance integration and develop more accurate measures to reveal the exact health benefits associated with the integration.

## Notes

Supplement 1. **eAppendix.** Supplemental Methods
eReferences

Supplement 2.
Data Sharing Statement

## References

[1] Reich MR, Harris J, Ikegami N, . Moving towards universal health coverage: lessons from 11 country studies. Lancet. 2016;387(10020):811-816. doi:10.1016/S0140-6736(15)60002-2 26299185

[2] Yip WC-M, Hsiao WC, Chen W, Hu S, Ma J, Maynard A. Early appraisal of China’s huge and complex health-care reforms. Lancet. 2012;379(9818):833-842. doi:10.1016/S0140-6736(11)61880-1 22386036

[3] Huang X, Wu B. Impact of urban-rural health insurance integration on health care: evidence from rural China. China Econ Rev. 2020;64:101543. doi:10.1016/j.chieco.2020.101543

[4] Chen Y, Jin GZ. Does health insurance coverage lead to better health and educational outcomes? evidence from rural China. J Health Econ. 2012;31(1):1-14. doi:10.1016/j.jhealeco.2011.11.001 22277282

[5] Huang F, Gan L. The impacts of China’s urban employee basic medical insurance on healthcare expenditures and health outcomes. Health Econ. 2017;26(2):149-163. doi:10.1002/hec.3281 26524988

[6] Lei X, Lin W. The New Cooperative Medical Scheme in rural China: does more coverage mean more service and better health? Health Econ. 2009;18(S2)(suppl 2):S25-S46. doi:10.1002/hec.1501 19551752

