# Supplementary material for: Association of Urban-Rural Health Insurance Integration With Health Outcomes Among Middle-aged and Older Adults in Rural China
Source: JAMA Netw Open. 2023 Apr 4;6(4):e237583. doi: 10.1001/jamanetworkopen.2023.7583 (PMC10074216; doi:10.1001/jamanetworkopen.2023.7583)
Supplement: Supplement 1. — eAppendix. Supplemental Methods eReferences [file jamanetwopen-e237583-s001.pdf]

Supplemental Online Content

Ye X, Wang Y. Association of urban-rural health insurance integration with health outcomes among middle-aged and older adults in rural China. *JAMA Netw Open*. 2023;6(4):e237583. doi:10.1001/jamanetworkopen.2023.7583

eAppendix. Supplemental Methods
eReferences

This supplemental material has been provided by the authors to give readers additional information about their work.

© 2023 Ye X et al. *JAMA Network Open.*

eAppendix. Supplemental Methods

Using the different time of implementing the urban-rural health insurance integration at the provincial level, a multivariate regression model by the staggered difference in difference (DID) method was used to study the associations between the integration and health.[1,2] The specification is shown below:

$$Y_{ijt} = \alpha + \beta INT_{jt} + \mu \mathrm{X}_{ijt} + \xi_p + \eta_t + \epsilon_{ijt}$$

where Y_{ijt} is the health outcome of individual i in the province j in the year t. INT_{jt} is the policy indicator, taking the value of 1 if the province has implemented the integration by year t, and 0 otherwise. X_{ijt} are control variables at the individual level. ξ_p and $\eta_t$is the province and year fixed effects to control variations across provinces and years. ϵ_{ijt} is the error term. In this study, we allow for spatial correlation and time l correlation in outcomes by clustering the error term ϵ_{ijt} at the at province-year level. The model reported as fitted provides as an output mean differences.

The DID approach was used on the premise that health outcomes in the treatment and control groups would have parallel trends without a policy shock.[3] Based on previous literature, we used a multi-period DID model (event study model) to test this hypothesis.[4,5] Multi-period DID is a generalized DID method that can test the hypothesis of parallel trends before an event occurs and examine the dynamic association of an event with health outcomes. We conducted multiple-period DID estimations for health outcomes and the parallel trends were also validated.

© 2023 Ye X et al. *JAMA Network Open.*

eReferences

1. Chen H, Ding Y, Tang L, Wang L. Impact of urban–rural medical insurance integration on consumption: Evidence from rural China. *Economic Analysis and Policy* 2022; **76**: 837-51.

2. Wang Z, Yin QE, Yu L. Real effects of share repurchases legalization on corporate behaviors. *Journal of Financial Economics* 2021; **140**(1): 197-219.

3. Marcus M, Sant'Anna PH. The role of parallel trends in event study settings: an application to environmental economics. *Journal of the Association of Environmental and Resource Economists* 2021; **8**(2): 235-75.

4. Cabral M, Geruso M, Mahoney N. Do larger health insurance subsidies benefit patients or producers? Evidence from Medicare Advantage. *American Economic Review* 2018; **108**(8): 2048-87.

5. Goodman-Bacon A. Public insurance and mortality: evidence from Medicaid implementation. *Journal of Political Economy* 2018; **126**(1): 216-62.

© 2023 Ye X et al. *JAMA Network Open.*
