# Supplementary material for: Association of Urban-Rural Health Insurance Integration With Health Outcomes Among Middle-aged and Older Adults in Rural China
Source: JAMA Netw Open. 2023 Apr 4;6(4):e237583. doi: 10.1001/jamanetworkopen.2023.7583 (PMC10074216; doi:10.1001/jamanetworkopen.2023.7583)
Supplement: Supplement 2. — Data Sharing Statement [file jamanetwopen-e237583-s002.pdf]

# Data Sharing Statement

Ye. Association of Urban-Rural Health Insurance Integration With Health Outcomes Among Middle-Aged and Older Adults in Rural China. *JAMA Netw Open*. Published April 04, 2023. doi:10.1001/jamanetworkopen.2023.7583

**Data**
**Data available:** No
